# Synergistic Effect of Banaba Leaf Extract and Policosanol (Raydel^®^) Ameliorate High Cholesterol and High Galactose-Diet Induced Adverse Events in Zebrafish

**DOI:** 10.3390/ph18060860

**Published:** 2025-06-09

**Authors:** Kyung-Hyun Cho, Sang Hyuk Lee, Yunki Lee, Ashutosh Bahuguna, Ji-Eun Kim, Krismala Djayanti, Cheolmin Jeon

**Affiliations:** Raydel Research Institute, Medical Innovation Complex, Daegu 41061, Republic of Korea

**Keywords:** corosolic acid, policosanol, dyslipidemia, galactose, 4-hydroxynoneal, inflammation, paraoxonase, senescence

## Abstract

**Background**: This study aimed to explore the therapeutic potential of a dietary regimen of banaba leaf extract (BNB), policosanol (PCO, Raydel^®^), and their combination (BNB+PCO), to mitigate high cholesterol (HC) and high galactose (HG) diet-induced dyslipidemia, hyperglycemia, oxidative stress, senescence, and organ damage in zebrafish (*Danio rerio*). **Methodology**: Zebrafish (*n* = 28/group) were fed with HC (4% *w*/*w*)+HG (30% *w*/*w*) or HC+HG supplemented either with BNB (0.1% *w*/*w*) or PCO (0.1% *w*/*w*) or BNB+PCO (0.1% *w*/*w* each). Following 6 weeks of dietary intervention, biochemical and histopathological examinations across the groups were performed. **Results**: Post 6 weeks of consumption, the BNB+PCO group exhibited a significant 40% decrease in body weight (BW) relative to the BW of the HC+HG group, while the BNB or PCO groups displayed nonsignificant changes in BW. Both BNB and PCO reduced HC+HG-induced dyslipidemia and hyperglycemia; however, co-administration (BNB+PCO) demonstrated a significantly greater therapeutic effect in countering these conditions compared to either BNB or PCO alone. A similar effect of the BNB+PCO combination was observed on the elevation of plasma sulfhydryl content, paraoxonase (PON), and ferric ion reduction activity (FRA), with notably ~1.2-times (*p* < 0.01) higher levels compared to their corresponding values observed in the BNB or PCO groups. Significantly diminished plasma AST, ALT, hepatic interleukin 6 (IL-6) levels, and fatty liver changes were observed in response to BNB+PCO, compared to either BNB or PCO alone. Also, BNB+PCO displayed a higher curative effect against HC+HG-induced impairment of tissue regeneration than BNB or PCO alone. A notable effect of BNB+PCO was perceived in protecting kidneys, testis, and ovary damage. Consistently, BNB+PCO showed a profound impact on mitigating HC+HG elevated reactive oxygen species (ROS) generation, apoptosis, cellular senescence, and accumulation of brain-binding lipid proteins (BLBPs) and 4-hydroxynoneal (4-HNE) in the brain. **Conclusions**: The findings highlight the synergistic effects of the BNB and PCO combination to mitigate the adversity posed by the consumption of the HC+HG diet.

## 1. Introduction

The consumption of high amounts of galactose is associated with detrimental effects in a variety of organs mediated by various events, including the formation of harmful metabolites such as galactitol [1] and advanced glycation end products (AGEs) [2]. Galactitol accumulation substantially affects cellular antioxidants and facilitates the production of reactive oxygen species (ROS) that eventually damage macromolecules like proteins and DNA and alter the integrity of the cell membrane [2,3]. Likewise, the interaction of AGEs with their receptor RAGE activates the nuclear factor kappa-B (NF-κB) signaling pathway, fostering oxidative stress and inflammatory responses [4]. Also, the interaction of AGEs with RAGE plays a pivotal role in regulating genes that contribute to the development of type-2 diabetes mellitus [4]. Besides the substantially adverse effects of galactose on vital organs like the kidney, liver, spleen, testis, thymus, and brain, it also contributed substantially to inducing cataracts, leading to vision loss and eye damage [5]. Galactose is one of the major agents responsible for premature aging [2], which triggers various cellular events, including senescence, sustained oxidative stress, and chronic inflammation, closely resembling the natural aging process [2].

High cholesterol [5] and a high-fat diet [6] provoke dyslipidemia, which negatively impacts the functionality of critical organs (e.g., liver, kidney, testes, and ovaries) [7,8,9] and has a notable effect on coronary heart disease [10]. Together, high cholesterol and high sugar regimens serve as a robust experimental model to induce metabolic disturbance, fatty liver, obesity, and insulin resistance, mirroring human disease pathology [11].

Various synthetic compounds and drugs have been explored against dyslipidemia, though their long-term consumption often emerged with certain adverse effects [12,13]. On the contrary, various natural products are found to be safe and effective against dyslipidemia [14] and other metabolic diseases [15]. Among the variety of natural products, policosanol gained substantial attention in countering dyslipidemia [16,17]. In particular, the effect of policosanol has been well described for the quality and quantity improvement in high-density lipoprotein (HDL) [18], a multifaceted molecule with a variety of beneficial properties. In a comparative study with patients of hypercholesterolemia and type 2 diabetes, policosanol emerged with better efficiency than the classical lipid-lowering agent (lovastatin) on the augmentation of HDL-C and minimizing the ratio of LDL-C/HDL-C [19], underscoring policosanol effectiveness over statins. Even more, the reports suggest that policosanol, in combination with classical lipid-lowering drugs (statin) effectively minimizes the statin-induced side effects [20,21]. Policosanol is a blend of long-chain aliphatic alcohols (LCAAs) with a general formula of CH_3_ (CH_2_)n CH_3_-OH (*n* = 22–32) [22,23] and can be obtained from a variety of sources originating from plants (e.g., rice bran, grapes, wheat germ, maize, and sugarcane) and animals (e.g., beeswax) [16,24,25]. The composition and amount of LCAA in policosanol vary considerably based on the source materials, origin of source material, and time of harvesting and extraction [16,26], which ultimately impacts the functionality of policosanol.

*Lagerstroemia speciosa* (banaba) is a widely grown plant in South Asian countries and is well-recognized for its distinct biological properties [27,28]. In particular, banaba leaves and their principal phytoconstituent corosolic acid exhibit multiple pharmacological activities, yet they are mainly recognized for their antidiabetic properties, mediated by several mechanisms that improve insulin resistance and insulin secretion [27,29]. As a key event, banaba (corosolic acid) modulates adenosine monophosphate-activated protein kinase (AMPK) via regulating the protein kinase B (Akt), insulin receptor substrate-1 (IRS-1), and liver kinase (LkB1), therefore, improving insulin sensitization and consequent effect [30]. In addition, banaba substantially affects the postprandial sugar level by inhibiting the activity of α-amylase and α-glucosidase [27,29].

Despite several studies highlighting the efficacy of policosanol [16,17] and banaba [27,29] in managing dyslipidemia and blood glucose levels, respectively, research exploring their combined therapeutic potential remains limited, particularly their joint effects on dyslipidemia, hyperglycemia, and associated adversity. This combined approach may offer a promising strategy to address dyslipidemia and hyperglycemia simultaneously. In a recent study, we tested the combined effect of banaba and policosanol and observed their synergistic implication in ameliorating streptozotocin-induced diabetic tissue regeneration and related complications in zebrafish [31]. Concerning these positive findings, we hypothesized that, in combination, policosanol and banaba, owing to their respective dyslipidemic and hypoglycemic properties, could provide an effective intervention for chronic metabolic disorders and their associated complications.

Given this, the present study aimed to investigate the comparative effect of 6 weeks of supplementation of banaba leaf extract (BNB), policosanol (PCO), and their combination (BNB+PCO) on high cholesterol (HC, 4% *w*/*w*) and high galactose (HG, 30% *w*/*w*) diet-induced dyslipidemia, hyperglycemia, oxidative stress, and impaired antioxidant parameters in zebrafish. Furthermore, this study explored the efficacy of these supplements in preventing cellular senescence and organ (liver, kidney, brain, testes, and ovaries) damage of zebrafish associated with the HC+HG diet.

The zebrafish was chosen as the experimental animal owing to its high genomic homology with humans, rendering it a valuable organism for preclinical research [32]. Zebrafish have been recognized as a canonical model for studying fertility [33], type 2 diabetes [34], dyslipidemia and associated disorders [35], toxicology, and drug discovery [36], owing to their mechanistic similarities with humans. Strikingly, zebrafish share key features of lipid metabolism with humans, including comparable receptors, lipoproteins, and critical enzymes involved in lipoprotein metabolism [37]. Unlike mice and rats, zebrafish possess cholesteryl ester transfer protein (CETP), a crucial component of human lipoprotein metabolism [37], further establishing zebrafish as an excellent preclinical model for lipoprotein-related research.

## 2. Results

### 2.1. Survival Analysis and Variation in Body Weight

**Figure 1:**
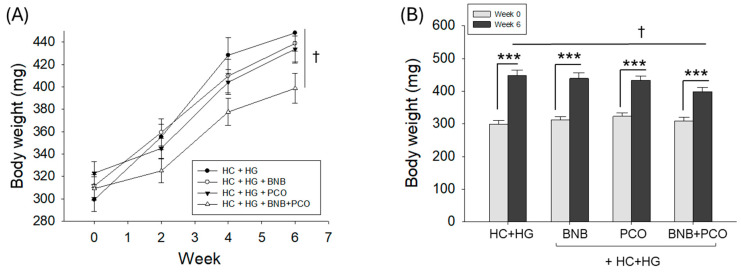
Effect of dietary intake of banaba (BNB, 0.1% *w*/*w*), policosanol (PCO, 0.1% *w*/*w*), and mixture of the banaba and policosanol (BNB+PCO, each 0.1% *w*/*w*) supplemented with high cholesterol (HC, 4% *w*/*w*) and high galactose (HG, 30% *w*/*w*) diet on the body weight of zebrafish. (**A**) Body weight changes over time (0–6 weeks), and (**B**) week 0 and week 6 body weights under respective dietary regimes. The ^†^ highlights the significant variance at *p* < 0.05 vs. HC+HG group, while *** represents the statistical difference between week 0 and week 6 among the respective groups.

A 100% survivability of zebrafish was observed among all the groups during 6 weeks of supplementation of the respective diets (Table 1). Contrary to this, a substantial variation in body weight (BW) was noticed across the different groups (Table 1 and Figure 1).

BW increased significantly (*p* < 0.001) in all groups after 6 weeks of supplementation compared to baseline (week 0). However, the most pronounced BW gain was observed in the HC+HG (control) group, which exhibited a 50% increase relative to week 0 (Table 1, Figure 1). In contrast, the BNB+PCO group showed the least increase in BW, approximately 29%. Supplementation with BNB or PCO alone resulted in intermediate BW gains of 41% and 34%, respectively.

When compared between the BW of 6 weeks, the BNB+PCO group (399 ± 13 mg) displayed a significantly (*p* < 0.05) lower BW weight compared to the HC+HG groups (448 ± 16 mg). Individually, 6 weeks of supplementation of BNB or PCO showed a reduced BW compared to the HC+HG groups; however, these changes are statistically non-significant (Table 1, Figure 1). These findings suggest that the combined supplementation of BNB+PCO is more effective in mitigating HC+HG-induced BW gain than either BNB or PCO alone.

### 2.2. Lipid Profiles in Plasma

**Figure 2:**
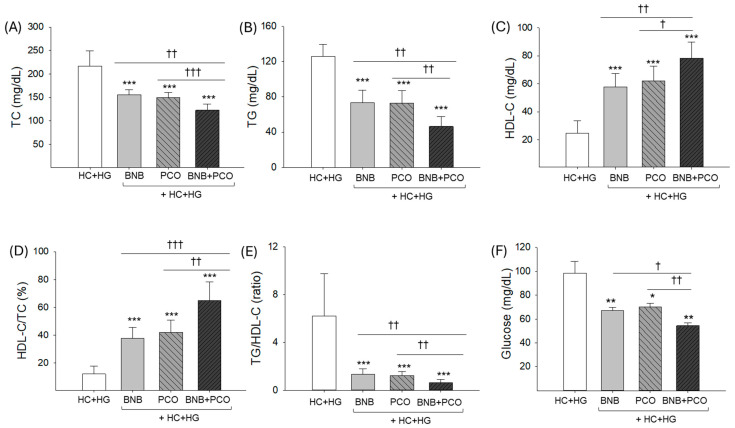
Comparison of blood lipid profile and glucose level after 6 weeks of consumption of banaba (BNB, 0.1% *w*/*w*), policosanol (PCO, 0.1% *w*/*w*), and mixture of the banaba and policosanol (BNB+PCO, each 0.1% *w*/*w*) supplemented with high cholesterol (HC, 4% *w*/*w*) and high galactose (HG, 30% *w*/*w*) diet. (**A**) Total cholesterol (TC), (**B**) triglycerides (TGs), (**C**) high-density lipoprotein cholesterol (HDL-C), (**D**) percentage ratio of HDL-C/TC, (**E**) ratio of TG/HDL-C, and (**F**) blood glucose level. The *, **, and *** underscore the statistical difference at *p* < 0.05, *p* < 0.01, and *p* < 0.001, respectively vs. HC+HG group. The ^†, ††^, and ^†††^ highlights statistical difference at *p* < 0.05, *p* < 0.01, and *p* < 0.001, vs. BNB+PCO.

As shown in Figure 2A,B, the HC+HG group showed the highest TC and TG levels of approximately 216.7 ± 32.2 mg/dL and 126.1 ± 13.2 mg/dL, respectively, while BNB+PCO group showed the lowest TC and TG levels of approximately 122.5 ± 13.1 mg/dL and 46.4 ± 11.3 mg/dL, respectively, which were 44% (*p* < 0.001) and 64% (*p* < 0.001) diminished relative to the HC+HG group. The BNB and PCO groups showed ~30% and ~40% lower TC and TG levels than those of the HC+HG group; nevertheless, in relation to BNB+PCO group, ~20% and ~50% elevated TC and TG levels were observed in the BNB and PCO groups. These results suggest that the co-consumption of BNB+PCO showed a significant effect on lowering TC and TG levels, rather than PCO or BNB alone.

As shown in Figure 2C,D, HC+HG group showed the lowest HDL-C level and HDL-C/TC (%) of approximately 24.6 ± 9.1 mg/dL and 12.1%, respectively, while the BNB+PCO group showed the highest HDL-C level and HDL-C/TC (%) of approximately 78.3 ± 11.3 mg/dL and 65%, respectively, which are 3.1-fold and 5.4-fold higher than those of the HC+HG group. The PCO and BNB groups showed 2.4~2.6-fold elevated HDL-C levels and 3.0~3.5-fold higher HDL-C/TC (%) than those of the HC+HG group. However, the PCO group and BNB group showed 21~27% and 36~44% lower HDL-C levels and HDL-C/TC (%) than those of the BNB+PCO group.

Concomitantly, the TG/HDL-C ratio was the highest and the lowest in the HC+HG group and PCO+BNB group, respectively (Figure 2E). These results suggest that the co-consumption of BNB+PCO showed a substantial effect in treating dyslipidemia by raising HDL-C and HDL-C/TC (%) and lowering the TG/HDL-C ratio compared with PCO or BNB alone.

As shown in Figure 2F, the HC+HG group showed the highest blood glucose level (98.5 ± 9.7 mg/dL), while the BNB+PCO group showed the lowest level (54.5 ± 2.9 mg/dL, *p* < 0.01). Glucose levels in the BNB and PCO groups (68–70 mg/dL) were ~30% lower than those in the HC+HG group, but ~25% more compared to the BNB+PCO group. These findings suggest that the co-consumption of PCO and BNB produces a synergistic glucose-lowering effect compared to the individual consumption of PCO or BNB.

### 2.3. Plasma Antioxidant Capacity

**Figure 3:**
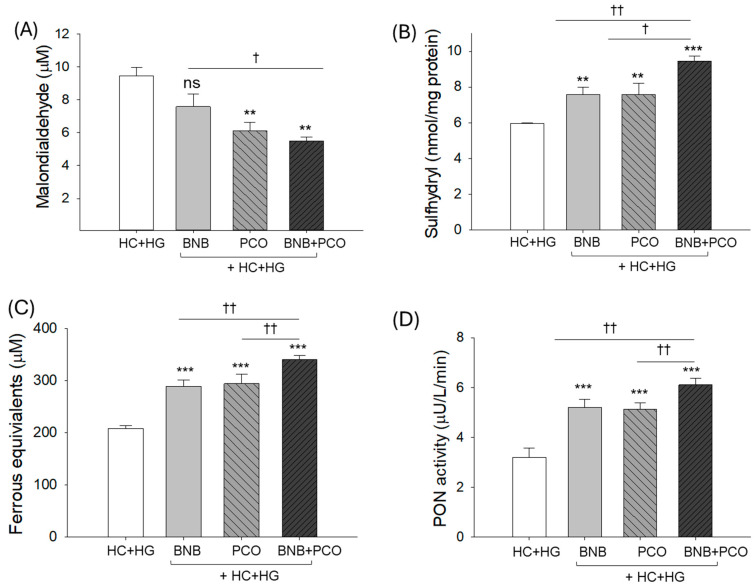
Comparison of oxidative variables and antioxidant abilities in zebrafish plasma after 6 weeks of consumption of banaba (BNB, 0.1% *w*/*w*), policosanol (PCO, 0.1% *w*/*w*) and mixture of the banaba and policosanol (BNB+PCO, each 0.1% *w*/*w*) supplemented with high cholesterol (HC, 4% *w*/*w*) and high galactose (HG, 30% *w*/*w*) diet. (**A**) Malondialdehyde level, (**B**) sulfhydryl content, (**C**) ferric ion reduction ability (FRA), and (**D**) paraoxonase (PON) activity. The ** and *** underscore the statistical difference at *p* < 0.01 and *p* < 0.001, respectively, vs. the HC+HG group. The ^†, ††^ and highlights statistical difference at *p* < 0.05, and *p* < 0.01, vs. BNB+PCO. The “ns” highlights the non-significant difference vs. HC+HG group.

The highest MDA level (9.5 ± 0.5 μM) was detected in the HC+HG supplemented group, which was reduced to 7.5 ± 0.8 μM and 6.1 ± 0.5 μM by the intake of BNB and PCO, respectively. However, the most promising outcomes with the lowest MDA level (5.5 ± 0.3 μM) were observed in the BNB+PCO supplemented group, which was notably 36.4%, 11.0%, and 70.8% lower than the MDA level detected in the BNB, PCO, and HC+HG supplemented groups, respectively (Figure 3A).

As shown in Figure 3B–D, the BNB+PCO group showed the highest sulfhydryl content, ferric ion reduction (FRA), and paraoxonase (PON) activity, approximately 1.6-, 1.6-, and 1.9-fold higher than the HC+HG group, respectively. Both BNB and PCO groups showed a substantial effect to elevate the HC+HG diminished sulfhydryl content, reflected by the ~1.3-fold higher sulfhydryl content than the HC+HG group. Similarly, the BNB and PCO groups displayed ~1.4- and 1.6-fold enhancements in the FRA and PON activities compared to the HC+HG group. However, compared to the combined effect of BNB+PCO, a significantly 13~20% inferior effect was displayed by BNB and PCO to elevate the HC+HG diminished sulfhydryl content, FRA, and PON activity. These findings indicate that the combined intake of PCO and BNB resulted in synergistic elevation of FRA, PON activity, and sulfhydryl contents in blood, compared with PCO and BNB alone.

### 2.4. Tail Fin Regeneration Speed and Morphology

**Figure 4:**
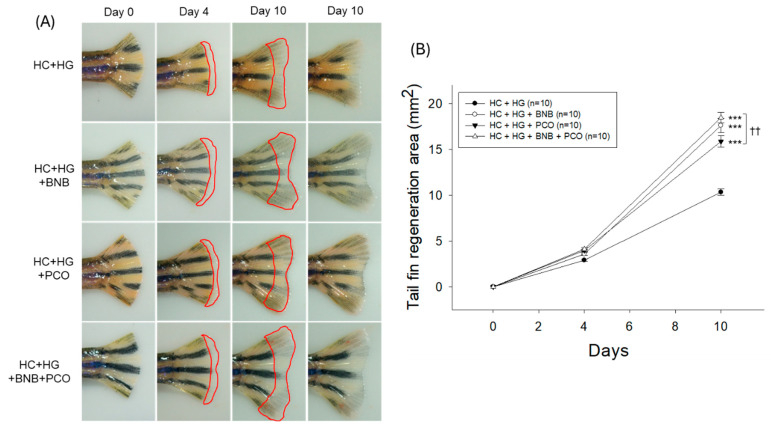
Comparison of amputated tail fin regeneration of zebrafish following consumption of banaba (BNB, 0.1% *w*/*w*), policosanol (PCO, 0.1% *w*/*w*), and mixture of the banaba and policosanol (BNB+PCO, each 0.1% *w*/*w*) supplemented with high cholesterol (HC, 4% *w*/*w*) and high galactose (HG, 30% *w*/*w*) diet. (**A**) Tail fin regeneration morphology during 10 days post-amputation. The regenerated tissue area is bordered by the red line. (**B**) Quantification of the regenerated tail fin area during 10 days post-amputation. The *** underscores the significant variance at *p* < 0.001 vs. group HC+HG. The ^††^ highlights statistical difference at *p* < 0.01 vs. BNB+PCO.

As shown in Figure 4A,B, a non-significant tail fin regeneration was observed between the groups up to 4 days post-amputation. With the progression of time up to 10 days post-amputation, the HC+HG group showed the lowest tissue regeneration (10.3 ± 0.4 mm^2^), while the BNB+PCO group exhibited the maximum level (18.5 ± 0.6 mm^2^) of the tail fin regeneration that was approximately 1.8-fold higher than the HC+HG group. Also, the BNB and PCO groups exhibited 1.5-fold and 1.7-fold higher tissue regenerations than the HC+HG group on day 10; nevertheless, they exhibited 5~14% lower tissue regeneration compared to the BNB+PCO group. These outcomes suggest that combined BNB and PCO intake significantly improved tissue regeneration relative to either PCO and BNB alone.

### 2.5. Hepatic Damage Markers in Plasma

**Figure 5:**
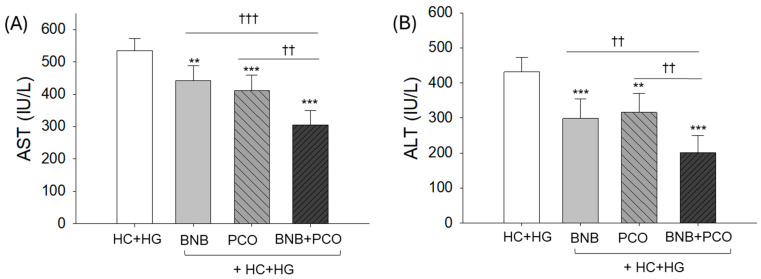
Comparison of hepatic enzymes (**A**) aspartate aminotransferase (AST) and (**B**) alanine aminotransferase (ALT) in zebrafish plasma after 6 weeks of consumption of banaba (BNB, 0.1% *w*/*w*), policosanol (PCO, 0.1% *w*/*w*) and a mixture of the banaba and policosanol (BNB+PCO, each 0.1%, *w*/*w*) supplemented with high cholesterol (HC, 4% *w*/*w*) and high galactose (HG, 30% *w*/*w*) diet. The ** and *** underscore the statistical difference at *p* < 0.01 and *p* < 0.001, respectively, vs. HC+HG group. The ^††^ and ^†††^ represent significant variance at *p* < 0.01 and *p* < 0.001, vs. BNB+PCO.

Figure 5 demonstrated that the HC+HG control group exhibited the maximum AST and ALT levels, approximately 534.8 ± 38.2 and 431.2 ± 41.5 IU/L, respectively, while BNB+PCO group showed the lowest AST (305.9 ± 43.6 IU/L) and ALT (201.1 ± 48.8 IU/L) levels of approximately 43% and 52% less (*p* < 0.001) than the HC+HG (control). Individually, both BNB and PCO groups showed 18~23% and 27~31% lower AST and ALT levels, respectively, than the HC+HG control group. Compared to the individual supplementation, a combined supplementation (BNB+PCO) showed 26% and 31% less AST and 37% and 33% less ALT than that of the PCO group and BNB group, respectively. These findings indicate that BNB and PCO act synergistically to mitigate HC+HG diet-induced hepatic damage, resulting in a severe alleviation of hepatic enzymes in plasma.

### 2.6. Histological and Immunohistochemical Analysis of Liver

**Figure 6:**
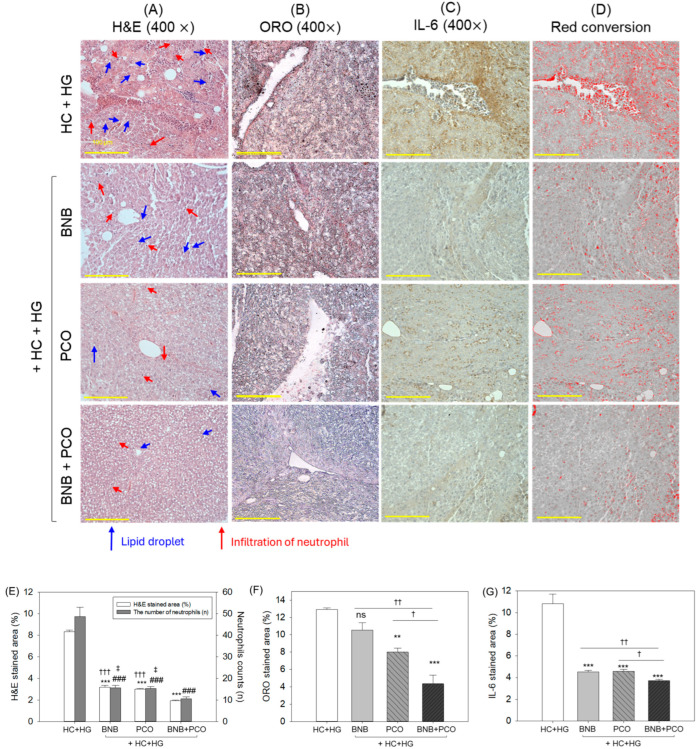
Histological analysis of hepatic tissue after 6 weeks of consumption of banaba (BNB, 0.1% *w*/*w*), policosanol (PCO, 0.1% *w*/*w*), and mixture of the banaba and policosanol (BNB+PCO, each 0.1%, *w*/*w*) supplemented with high cholesterol (HC, 4% *w*/*w*) and high galactose (HG, 30% *w*/*w*) diet. (**A**) Hematoxylin and eosin (H&E) staining, (**B**) oil red O (ORO) staining, (**C**) immunohistochemical analysis to detect interleukin 6 (IL-6), and (**D**) red conversion of the IL-6-stained area (to improve the visibility) using brown color threshold value 20–120 employing Image J software (https://imagej.net/ij, 1.53 version, accessed on 6 June 2023). (**E**) Quantification of H&E-stained area and neutrophil counts. (**F**,**G**) Quantification of ORO, and IL-6-stained area. The ** and *** underscore the significant variance at *p* < 0.01, *p* < 0.001 (for H&E, ORO, and IL-6-stained area), while ^###^ underscore the statistical difference at *p* < 0.001 (for neutrophil counts) vs. HC+HG group. The ^†^, ^††^, and ^†††^ highlight statistical differences at *p* < 0.05, *p* < 0.01, and *p* < 0.001 (for H&E and IL-6-stained area), while ^‡^ represents the statistical difference at *p* < 0.05 (for neutrophil counts) vs. BNB+PCO group. The “ns ” highlights the non-significant difference vs. HC+HG group.

As depicted in Figure 6, the HC+HG group had the most extensive H&E-stained area with dense neutrophil infiltration; in contrast, the BNB+PCO group showed the least H&E-stained area and neutrophil presence, representing 78% reduction compared to the HC+HG group. The BNB and PCO alone also showed a substantial effect against HC+HG-induced events endurance by ~68% fewer neutrophil counts than the HC+HG group (Figure 6A,E). However, compared to the BNB and PCO alone groups, ~30% lower neutrophil counts were observed in the BNB+PCO supplemented group, suggesting that the combination of PCO and BNB resulted in profound inhibition of hepatic inflammation.

The highest ORO-stained area corresponding to hepatic fat accumulation was noted in the HC+HG group, which was markedly altered by the intake of BNB and PCO, reflected by 18% and 38% lower ORO-stained area among the corresponding groups relative to the HC+HG group (Figure 6B,F). Nonetheless, the combination of BNB+PCO emerged with the most notable outcomes, with a significant 59% and 45% reduction in ORO-stained area compared to the BNB and PCO alone supplemented groups.

As depicted in Figure 6C,D,G, immunohistochemical analysis indicated that the HC+HG group exhibited the utmost IL-6-stained area (approximately 10.8%), whereas the BNB+PCO group displayed the lowest area (approximately 3.7%). Also, BNB and PCO individually prevent HC+HG-induced IL-6 production; however, the effect is almost 20% inferior to the effect of BNB+PCO on IL-6 production, underscoring that the co-consumption of BNB and PCO have a substantially higher effect to inhibit hepatic inflammation.

### 2.7. Histological Analysis of Brain

**Figure 7:**
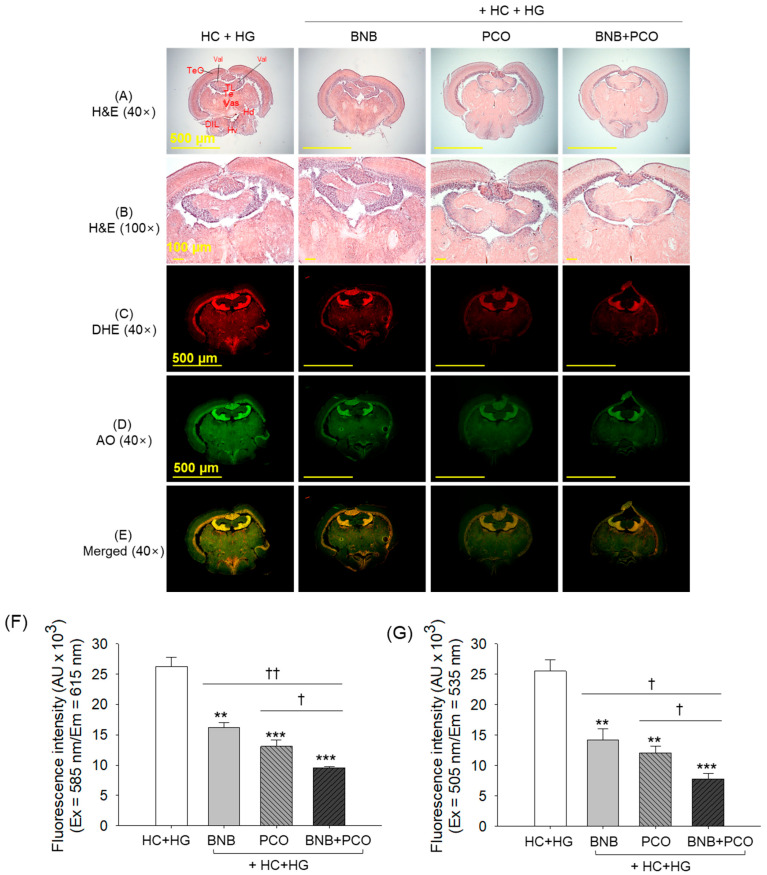
Brain histology after 6 weeks of consumption of banaba (BNB, 0.1% *w*/*w*), policosanol (PCO, 0.1% *w*/*w*), and mixture of the banaba and policosanol (BNB+PCO, each 0.1% *w*/*w*) supplemented with high cholesterol (HC, 4% *w*/*w*) and high galactose (HG, 30% *w*/*w*) diet. (**A**,**B**) Hematoxylin and eosin (H&E) staining at 40× and 100× magnification, respectively. (**C**) Dihydroethidium (DHE) staining. (**D**) Acridine orange (AO) staining and (**E**) merge images of DHE and AO staining. (**F**,**G**) Quantification of DHE and AO fluorescent intensities. The ** and *** underscore the significant variance at *p* < 0.01 and *p* < 0.001, vs. HC+HG group. The ^†^ and ^††^ highlight significant differences at *p* < 0.05 and *p* < 0.01, vs. BNB+PCO.

The outcomes of the H&E staining, as depicted in Figure 7, displayed non-substantial changes in the brain morphology across the groups concerning the vacuolation and mononuclear cells, featuring a transparent zone in the tectum optic (TeO) and periventricular grey zone (PGZ). Also, no presence of hyperemia was detected among the different groups consuming their respective diets.

Despite the non-substantial morphological alterations among the groups, the DHE and AO staining (Figure 7C–G) showed the most vigorous red and green fluorescent intensities in the HC+HG groups that account for the 1.5~2.0-times and 1.8~2.1-times elevation compared to BNB and PCO groups, respectively, suggesting severe ROS production and apoptosis in the brain of HC+HG supplemented zebrafish. However, compared to the individual supplementation of the PCO and BNB, combined supplementation of BNB+PCO displayed a more pronounced effect to mitigate the HC+HG-triggered ROS production and apoptosis manifested by the 1.4-times and 1.7-times reduced DHE fluorescent intensities and the 1.6 times and 1.8 times attenuated AO fluorescent intensities, respectively, compared to the PCO and BNB supplemented groups.

### 2.8. Cellular Senescence and Oxidative Stress in the Brain

**Figure 8:**
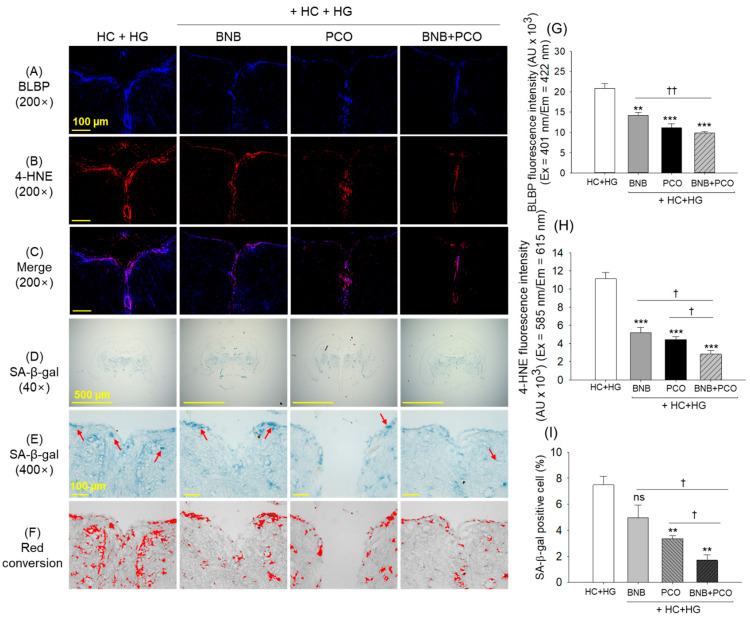
Comparison of brain lipid binding protein (BLBP), 4-hydroxynoneal (4-HNE) levels and cellular senescence in the zebrafish brain consuming banaba (BNB, 0.1% *w*/*w*), policosanol (PCO, 0.1% *w*/*w*), and mixture of the banaba and policosanol (BNB+PCO, each 0.1% *w*/*w*) amalgamated in high cholesterol (HC, 4% *w*/*w*) and high galactose (HG, 30% *w*/*w*) diet. (**A**,**B**) immunohistochemical (IHC) staining for the detection of brain lipid binding proteins (BLBPs) and lipid oxidize product 4-hydroxynoneal (4-HNE), (**C**) merged images obtained from BLBP and 4-HNE staining. (**D**,**E**) Senescent associated β-galactosidase (SA-β-Gal) staining at 40× and 100× magnification, respectively, the red arrow indicates senescent-positive cells, (**F**) red conversion of SA-β-Gal stained area (to improve the visibility) determined at blue color threshold value 0–120 employing Image J software. (**G**,**H**) Assessment of BLBP and 4-HNE fluorescent intensities, respectively. (**I**) Assessment of SA-β-Gal positive cells. The **, and *** underscore the significant variance at *p* < 0.01, and *p* < 0.001, vs. HC+HG group. The ^†^ and ^††^ highlight significant variance at *p* < 0.05 and *p* < 0.01 vs. BNB+PCO. The “ns” highlights the non-significant difference.

The immunohistochemical (IHC) outcomes revealed the high prevalence of brain-binding lipid proteins (BLBPs) and the oxidized lipids 4-hydroxynonenal (4-HNE) in the brain section precisely around vascular lacuna of area postrema (Vas) for the HC+HG fed group (Figure 8). The individual administration of BNB and PCO effectively reduced the BLBP accumulation in the brain, as depicted by a 1.5-fold and 1.9-fold diminished fluorescent intensities corresponding to the HC+HG supplemented group. Likewise, a 2.1-fold and 2.5-fold reduced 4-HNE accumulation was apparent in the BNB and PCO groups against the HC+HG group. Intriguingly, the combined intake of BNB+PCO displayed the remarkably highest effect in preventing the BLBP and 4-HNE buildup in the brain. As described in Figure 8A–C,G,H, the least BLBP and 4-HNE levels, which were 2.2-fold and 3.9-fold lower than their respective level in the HC+HG group, were observed in the BNB+PCO supplemented group. In comparison to the alone supplementation of PCO and BNB, a significant 1.2~1.5-fold and 1.6~1.8-fold reduced BLBP and 4-HNE accumulation, respectively, were observed in the BNB+PCO supplemented group, underlining the functional superiority of BNB+PCO combination to mitigate the HC+HG triggered adverse events in the brain.

The SA-β-gal staining of the brain section (Figure 8D–F,I) revealed the highest blue stained area in the HC+HG group, corresponding to 7.5% of SA-β-gal positive cells mainly in the Vas region of the brain cortex. In contrast, the BNB+PCO group showed the least blue-stained area, approximately 1.7%, which was 78% lower than the SA-β-gal-stained area that appeared in the HC+HG group. The individual supplementation of PCO and BNB showed 54% (*p* < 0.01) and 34% lower SA-β-gal positive cells than the HC+HG group, respectively. These results suggest that the co-consumption of PCO and BNB exerted synergistic effects to prevent cellular senescence in the brain under the HC+HG diet.

### 2.9. Histological Analysis of Kidney, Ovary and Testis

**Figure 9:**
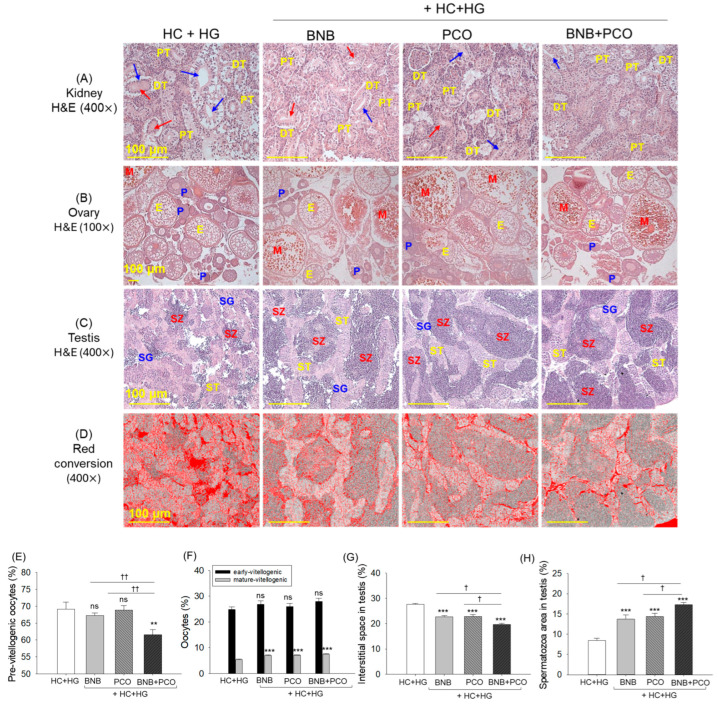
Histological analysis of kidney, ovary, and testis of zebrafish post 6 weeks of consumption of banaba (BNB, 0.1% *w*/*w*), policosanol (PCO, 0.1% *w*/*w*) and a mixture of the banaba and policosanol (BNB+PCO, each 0.1% *w*/*w*) amalgamated in high cholesterol (HC, 4% *w*/*w*) and high galactose (HG, 30% *w*/*w*) diet. (**A**–**C**) Hematoxylin and eosin (H&E) staining of the kidney, ovary, and testis, respectively. Arrows blue and red depict the elevated tubular lumen and luminal debris, respectively. PT and DT are abbreviated for proximal and distal tubule, while E, P, and M depict the early, pre, and mature oocytes. In the testis section, SG, ST, and SZ are abbreviations for spermatogonia, spermatocytes, and spermatozoa, respectively. (**D**) Red conversion of the testicular interstitial space (to enhance the visibility) employing the Image J software at the white color threshold value 220–255. Scale bar, 100 μm. (**E**) Previtellogenic oocytes count in the ovary section. (**F**) Oocytes (early and mature) count in the testis section. (**G**,**H**) percentage quantification of interstitial space and spermatozoa count in the testis. The ** and *** underscore the statistical difference at *p* < 0.01 and *p* < 0.001 vs. HC+HG group. The ^†^ and ^††^ highlight the significant differences at *p* < 0.05 and *p* < 0.01 vs. BNB+PCO. The “ns” highlights the non-significant difference.

The kidney histology of the HC+HG group revealed disorganized and sparsely populated proximal and distal tubules with broadened tubular lumen and frequent occurrence of luminal debris in the tubular cast (Figure 9A). In contrast, a substantial effect of BNB, PCO, and their combination (BNB+PCO) was observed to restore the HC+HG-induced pathological changes in the kidney by substantially preventing the dilation of tubular lumen and luminal debris in the tubular cast. However, compared to the BNB+PCO group, the occasional presence of cellular debris in the luminal cast and elevated tubular lumen was noticed in the BNB and PCO groups.

The H&E staining of the ovary section revealed the highest pre-vitellogenic oocyte (~70%) and the least mature oocyte counts (~70%) in the HC+HG group that was significantly reduced up to 64.2% following BNB+PCO supplementation (Figure 9B,E,F). Unlike the effect of BNB+PCO, the individual supplementation of BNB or PCO failed to alter the HC+HG-changed pre-vitellogenic oocyte counts; however, both BNB and PCO substantially improved the mature oocyte counts, which is statistically similar to the mature oocyte counts observed in the BNB+PCO supplemented group.

The H&E staining of the testis section displayed the loosely arranged seminiferous tubules, heightened interstitial space between the seminiferous tubules, and reduced spermatozoa in the HC+HG consumed groups (Figure 9C,D,G,H). These changes were substantially prevented by the consumption of BNB, PCO, and their combination (BNB+PCO). Notably, the combination of BNB+PCO displayed an appreciably higher protective effect compared to their individual supplementation, evident by the ~14% reduction in interstitial space between the seminiferous tubules and ~23% higher spermatozoa counts compared to the BNB and PCO supplemented groups, respectively.

## 3. Discussion

While numerous natural materials have demonstrated possibilities as lipid- and glucose-lowering agents [38,39], policosanol’s established efficacy as a lipid-lowering nutraceutical (approved in over 25 counties) [40] and promising glucose-regulating effect of banaba leaves/corosolic acid [27,29] formed the basic rationale for selecting these materials over others. The present research utilized zebrafish as a model organism due to its high genome similarities to humans and its ability to replicate the pathophysiology of many human diseases; consequently, the outcomes obtained from the zebrafish can be mimicked when prompted in human trials. Additionally, previous studies have demonstrated that zebrafish-based screening of small molecules has efficiently translated into clinical research, validating its suitability for preclinical investigations [32].

The adverse effects of high cholesterol and high sugar composition are well described for obesity, metabolic stress, and cardiovascular diseases [6,41]. Under the presence of high sugar, non-enzymatic protein glycation proceeds, leading to the formation of several toxic AGEs responsible for various toxicogenic effects [42]. The HC+HG-supplemented diets served as an excellent dietary model to induce obesity, insulin resistance, dyslipidemia, and vital organ damage. Herein, a 6-week consumption of an HC+HG diet displayed a substantial elevation in the BW of zebrafish, which remains nearly unchanged following either BNB or PCO supplementation. However, in response to the BNB+PCO supplementation, a significant drift in HC+HG-induced BW enhancement was noticed. Despite reports of anti-obesity activity for both PCO [16] and BNB [27], neither of them alone proved significantly effective in mitigating BW gain under prolonged HC+HG exposure. However, their combined use (BNB+PCO) resulted in a reduction in body weight, highlighting the synergistic effect of banaba in combination with policosanol in managing HC+HG-induced body weight gain. Nevertheless, extended consumption studies (>6 weeks) using various obesity-inducing models are needed to confirm the versatile anti-obesity potential of BNB+PCO.

The accumulating literature suggests the protective effect of banaba (due to its main component, corosolic acid) and policosanol against dyslipidemia [43,44]. In particular, policosanol has been recognized to alter the 3-hydroxy-3-methyl-glutaryl-coenzyme A reductase (HMG-CoA) (a key regulatory enzyme of cholesterol biosynthesis), consequently impacting cholesterol level [45]. The effect of policosanol in inhibiting the CETP and the expression of the major HDL protein apoA-I [18] has been recognized as an important event in the elevation of HDL-C level. Banaba has been documented to elevate HDL-C levels [43]; however, the mechanism behind this is not known. Corroborating with the earlier findings, we have noticed a diminished TC, TG, and elevated HDL-C level in response to BNB and PCO supplementation, which was substantially improved by the combination of BNB+PCO. Herein, we speculate that BNB+PCO worked in a synergetic fashion, contrary to their individual supplementation, towards the inhibition of CETP activity and enhancing apoA-I expression, resulting in the substantial rise in HDL-C level and mitigation of the HC+HG triggered dyslipidemia. Nevertheless, the precise mechanisms underlining the dyslipidemic effect of BNB+PCO co-supplementation warrant further investigation.

A galactose + high-fat diet and oxidative stress contribute to the onset of insulin resistance, and the inhibition of insulin secretion leads to hyperglycemia [46,47]. Policosanol’s impact on insulin sensitization and insulin secretion has been recognized via the AMPK and PI3/Akt signaling pathways [48]. Similarly, banaba (owing to the presence of corosolic acid) induced a variety of events, including modulation of AMPK and Akt signaling pathways and insulin receptor substance-I phosphorylation leading to hypoglycemia [30,49]. Following the above-mentioned events, banaba, in combination with policosanol (BNB+PCO), works efficiently and leads to a pronounced glucose-lowering effect compared to either BNB or PCO alone.

The HC+HG provided lucrative conditions to induce oxidative stress, marked by a high prevalence of MDA, diminished sulfhydryl content, and compromised FRA and PON activities. The HC+HG disturbed MDA, sulfhydryl level, FRA, and PON activities were substantially altered in response to BNB+PCO, which was much better than the individual effects exerted by BNB or PCO, documenting a positive impact of the combination to mitigate the oxidative challenge. High-sugar diets are associated with the production of AGEs that are well known for the induction of ROS, oxidative stress, inflammation, and disturbed redox homeostasis [50]. The positive role of banaba in the direct scavenging of free radicals and the modulation of cellular antioxidants has been described as a defensive mechanism against a variety of stresses [51,52,53], including diabetic conditions. Likewise, a suppressive effect of policosanol on ROS production and protein glycation has been noticed as a defensive shield against a variety of stresses, including severe toxicity posed by CML [54]. It is apparent that the combination of distinct activities of BNB and PCO working in a joint manner leading to synergistic amelioration of HC+HG provoked oxidative turbulence and antioxidant parameters.

The substantial effect of banaba and policosanol that was notably enhanced in their combination (BNB+PCO) was noticed in the regeneration of amputated tail fins under the hostile environment of HC+HG. A substantially higher effect of BNB+PCO compared to either BNB or PCO to curtail the HC+HG elevated glucose level might be a key factor for better tissue regeneration activity. The postulation is supported by the earlier reports outlining the provocatory effect of high glucose on tissue inflammation and vascular endothelial growth factors that eventually halted wound healing and tissue regeneration [55]. Also, the high sugar environment facilitates the production of AGEs that induce ROS generation, inflammation, and apoptosis in fibroblast and keratinocytes and hinder tissue regeneration [56]. The antiglycation effect of policosanol [54] and the antioxidant and anti-inflammatory effect of banaba [28] work cooperatively and facilitate higher tail fin regeneration in response to BNB+PCO supplementation compared with their individual effects.

Galactose favors AGE formation, heightened oxidative stress, and induced production of proinflammatory cytokines (such as IL-6 and TNF-α) [57] that prompted the liver damage. Consistent with prior reports, HC+HG treatment significantly induced fatty liver changes and hepatic inflammation, characterized by elevated neutrophil infiltration and IL-6 generation. A combination of BNB+PCO was found effective in altering the HC+HG-induced detrimental effect in the liver better than their individual effects. The antioxidant and anti-inflammatory properties of banaba [28] and the ability of policosanol to suppress ROS generation, inflammation, and protein glycation [54] represent key defensive mechanisms against HC+HG-induced events. In addition, both policosanol (due to LCAA, hexacosanol) [45] and banaba (corosolic acid) [58] were found effective in upregulating, which plays a crucial role in mitigating fatty liver changes, as autophagy has been directly implicated in the pathogenesis of fatty liver diseases [45]. The combination of BNB+PCO appeared to act synergistically, resulting in enhanced hepatoprotective effects compared to the effects observed with either BNB or PCO alone. The histological outcomes of the higher hepatoprotective effect of the BNB+PCO are corroborated with the outcomes of the plasma hepatic function biomarkers, where substantially lower levels of AST and ALT were noticed in the BNB+PCO group relative to the groups receiving individual supplementation of BNB or PCO.

The dyslipidemia has been described to damage kidney and reproductive organs [59,60]. Similarly, high galactose intake is also linked with kidney [61] and reproductive organ impairment [62]. In line with earlier studies, we have observed kidney and reproductive organ impairment in response to the HC+HG consumption that was substantially reverted by the supplementation of BNB+PCO. The induction of oxidative stress and AGE accumulation have been recognized as key events of kidney damage [63]. Therefore, the substance exhibiting strong antioxidant [64] and antiglycation [61] abilities has shown promising prospective against kidney damage. The antioxidant nature of banaba [28,51,52] and the antiglycation effect of policosanol [53] are the crucial factors that work cooperatively to facilitate a higher renal-protective effect when used in combination (BNB+PCO), contrary to their solitary supplementation. The histological outcomes of better kidney health in the BNB+PCO group align with the findings of elevated plasma sulfhydryl levels in response to the BNB+PCO supplementation. Notably, reduced sulfhydryl levels have been associated with kidney disease and are considered a marker of impaired kidney function [65].

The adverse effect of a high-fat [66] and galactose [67] diet on testicular damage has been described. The consumption of high galactose prompts ROS production and disrupts antioxidant homeostasis, leading to testis [67] and ovary impairment [68]. Likewise, sugar-provoked AGE generation has been implicated in reproductive organ dysfunction by the induction of oxidative stress [69]. Compounds with antioxidant properties have been mentioned to prevent testicular [67] and ovary damage [68] posed by external stress. Due to the antioxidant nature of banaba [28,51,52] and the antiglycation effects of policosanol [54] their combination (BNB+PCO) displayed an effective role in mitigating the HC+HG-induced detrimental effects in the reproductive organs.

A definite effect of galactose on brain damage and aging has been described via the accumulation of AGEs [70], oxidative stress [71], and compromised cellular antioxidants [72]. Herein, we have noticed a substantial ROS generation, apoptosis, senescence, and expression of brain lipid binding protein (also known as fatty acid-binding proteins) in response to HC+HG consumption. Notably, the overexpression of BLBP was found detrimental to prospective neuroprotection and ischemic stroke [73]. Similarly, HC+HG intake leads to the higher buildup of 4-HNE, an important lipid peroxidation product associated with oxidative stress that prompts neuronal cell death by the involvement of caspase 3 mediate apoptosis [74]. The inhibitory effect of PCO on the ROS generation, apoptosis, and substantial antioxidants, anti-inflammatory and anti-apoptotic properties of BNB working in a joint manner leads to higher efficacy to prevent HC+HG induced brain damage in the BNB+PCO consumed group. Earlier studies corroborate this notion, demonstrating the healing effect of antioxidants in reducing 4-HNE generation and associated detrimental effects [74]. Besides direct antioxidant and anti-apoptotic effects in the brain, a substantial liver and kidney protective effect of BNB+PCO is a reason for better brain health. The notion is supported by the literature describing a liver–brain [75] and kidney–brain axis [76]. Heavy deposition of fat in the liver alters the blood-brain barrier (BBB) permeability and facilitates the buildup of toxic material and inflammatory cells in the brain, giving rise to brain damage [77]. Oxidative insult and chronic kidney disease impact on brain and neuro-psychiatric disorders highlight the kidney–brain axis [76]. BNB+PCO substantially protects the liver and kidney, thus affirmatively modulating the liver–brain and kidney–brain axis and protecting the brain from the detrimental effect of HC+HG.

## 4. Materials and Methods

### 4.1. Plant Materials and Antibodies

Ethanolic extract of the banaba leaves (batch no. UO/LSD-1825/02/23-24) was purchased from the Umalaxmi Organics Pvt. Ltd. Jodhpur, Rajasthan, India. The Cuban sugarcane wax extracted policosanol (batch no. 310030324) was complementary, provided by the Raydel^®^ Pty. Ltd. (Sydney, Australia) that harbors a blend of eight long-chain aliphatic alcohols (LCAAs) of carbon chain C24 to C34. A certificate of analysis with specifications for banaba extract and policosanol is provided in Supplementary Tables S1 and S2, respectively. Primary antibodies specific against brain lipid binding protein (BLBP, ABN14) were procured from Sigma Aldrich (St. Louis, MO, USA), while the antibodies targeting 4-hydroxynonenal (4-HNE, ab45506) and interleukin 6 (IL-6, ab9324) were purchased from Abcam (Cambridge, UK). All chemicals and reagents were of analytical grade and utilized as supplied. Detailed specifications of the materials are provided in Supplementary Material Section S1.

### 4.2. Zebrafish Culturing

A wild type of zebrafish (*Dania rerio*, AB strain) was maintained at 28 °C water temperature in the tank equipped with a circulating water system. The zebrafish were maintained at a consistent light–dark cycle of 14 hr and 10 hr, respectively. Zebrafish were cultured following the compliance with the Animal Care and Use adopted by Raydel Research Institute (approval code RRI-23-2007, approval date 27 July 2023) and fed with normal tetrabit (ND, Tetrabit Gmbh D49304).

### 4.3. Specialized Diet Preparation

The normal tetrabit (ND) was supplemented with high cholesterol (HC, final 4% *w*/*w*) together with high galactose (HG, final 30% *w*/*w*) to make the HC+HG diet. The HC+HG was blended with banaba extract (BNB, final 0.1% *w*/*w*) or policosanol (PCO, final 0.1% *w*/*w*) or banaba+policosanol combination (BNB+PCO, final 0.1% *w*/*w* each) to make three specialized dietary formulations, and marked as HC+HG+BNB, HC+HG+PCO, and HC+HG+BNB+PCO, respectively. The detailed composition and proportions of dietary ingredients for the specified diets are listed in Table 2.

### 4.4. Feeding of the Zebrafish

**Figure 10:**
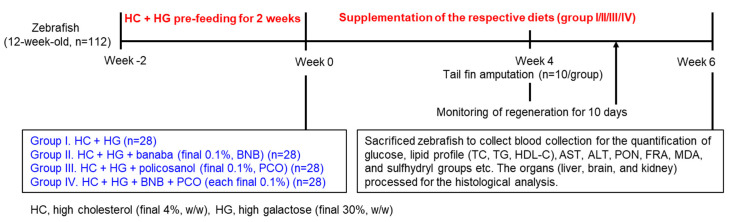
The study plan of a 6-week dietary intervention in zebrafish, incorporating a high-cholesterol (HC) and high-glucose (HG) diet infused with banaba (BNB), policosanol (PCO), or a combination of both (BNB+PCO).

Young (12-week-old, *n* = 112) zebrafish were maintained for 2 weeks in the HC+HG diet to induce metabolic stress. Further, the zebrafish were randomly allocated into 4 groups (*n* = 28 in each group) and fed either with HC+HG (group I), HC+HG+BNB (group II), HC+HG+PCO (group III), or HC+HG+BNB+PCO (group IV) for 6 weeks (Figure 10). For each group (*n* = 28), zebrafish were assigned to four separate tanks (*n* = 7/tank) and supplemented with the specified diets for 6 weeks. Zebrafish in each tank (*n* = 7) received 70 mg of the respective diets, administered twice daily [morning (~9.0 am) and evening (~6.0 pm)], cumulating 140 mg/tank (~equivalent to 20 mg/day/zebrafish). During 6 weeks of supplementation of the respective diets, survivability and body weight (BW) of the zebrafish across all the groups were examined.

### 4.5. Tail Fin Amputation

After four weeks’ intake of the respective diet, zebrafish (*n* = 10 from each group) were separated and transferred into a tank containing 2-phenoxyethanol solution (0.1%) to anesthetize. The anesthetized zebrafish tail fin was amputated adjacent to the dermal rays using the surgical blade. The regeneration of the amputated tail fin was monitored under the microscope at 0 days, 4 days, and 10 days. The images of the respective days were captured and processed to quantify the regenerated tail fin area using Image J software (version 1.53, https://imagej.net/ij, assessed on 16 June 2023).

### 4.6. Collection of Blood and Organs

Following 6 weeks of supplementation, zebrafish from all experimental groups were euthanized using hypothermic shock, and blood was immediately collected. Prior to the blood collection, the zebrafish were fasted overnight (~14 h). To reduce potential variations due to circadian rhythms or feeding effects, all samples were taken on the same day between 9 am and 10 am.

For the blood collection, 7 zebrafish/tanks of a specified group were sacrificed, and ~2–5 μL blood from each zebrafish was collected and pooled. The pooled blood from 7 zebrafish/tank (for the specified group) was mixed with phosphate-buffered saline (PBS)-ethylenediaminetetraacetic acid (EDTA, final concentration, 1 mM) in 2:3 (vol/vol) and centrifuged to obtain the plasma. The different organs (liver, kidney, brain, testis, and ovary) were surgically removed and preserved in 10% formalin solution for further analysis.

### 4.7. Blood Analysis for the Quantification of Lipoprotein, Hepatic Function Biomarkers and Glucose Level

Blood total cholesterol (TC), triglycerides (TGs), high-density lipoprotein cholesterol (HDL-C), and hepatic function biomarkers aspartate aminotransferase (AST) and alanine aminotransferase (ALT) were quantified using commercial kits following the instruction in accordance with the manufacturer’s guidelines. A comprehensive methodological approach is outlined in the Supplementary Method Section S2. Blood glucose concentration was measured using an automated digital glucometer (AccuCheck, Roche, Basel, Switzerland).

### 4.8. Evaluation of Malondialdehyde (MDA), Sulfhydryl Content and Antioxidant Parameters

The blood MDA level was quantified using the earlier described method [31]. Briefly, 20 μL plasma (1 mg mL^−1^ equivalent protein) was mixed with 50 μL and 100 μL of trichloroacetic acid (0.2 g mL^−1^) and thiobarbituric acid (6.7 mg mL^−1^), respectively. The content was incubated at 95 °C for 10 min, following the recording of absorbance at 560 nm.

The sulfhydryl content was quantified using a spectroscopic method following the earlier described method [31]. Briefly, 50 μL plasma (1 mg mL^−1^ equivalent protein) was mixed with 50 μL of 5,5-dithio-bis-(-2-nitrobenzoic acid) (4 mg mL^−1^) followed by 120 min incubation at room temperature (RT) and determination of the absorbance at 412 nm. The molar absorbance coefficient (ε = 136 × 10^2^ M^−1^cm^−1^) of the reaction product (5-thiol-2-nitrobenzoic acid), was used to express the results in mmol/mg protein.

To assess ferric ion reduction (FRA) capacity, 20 μL of the plasma (1 mg mL^−1^ equivalent protein) was mixed with 180 μL of FRA reagent [31]. After incubating the mixture at RT for 60 min, absorbance was measured at 593 nm. The results were quantified in μM ferric equivalents based on a ferrous sulfate standard curve.

The paraoxonase activity in the plasma was determined by the earlier described method [31]. A detailed procedure is provided as a Supplementary Method Section S3.

### 4.9. Histological Analysis

The 7 μm thick tissue section of the liver, kidney, brain, ovary, and testis was obtained from the cryo-sectioning using cryo-microtome (Leica Biosystem, Nussloch, Germany). The respective tissue sections (7 μm thick) were processed for the hematoxylin and eosin (H&E) staining following the earlier described method [78].

In the liver tissue section, oil red O (ORO) staining was implemented to observe the fatty liver changes following the earlier described method [24]. Briefly, 500 μL of ORO solution was spread over the liver section (7 μm thick) and incubated for 5 min at 60 °C followed by washing with 60% isopropanol and visualization under the microscope.

### 4.10. Immunohistochemical (IHC) Staining

Interleukin 6 (IL-6) production in the liver and the expression of brain-binding lipid protein (BLBP), and 4-hydroxynonenal (4-HNE) in the brain were examined by the IHC staining using the primary antibodies against the specific targets. For the IL-6 detection, hepatic tissue (7 μm thick) was covered with anti-IL-6 antibody (Abcam ab9324) and incubated in a cool moist environment for 16 hr. An EnVision+system HRP polymer kit (Dako, Glostrup, Denmark) was utilized to develop the section.

For the detection of BLBP and 4-HNE expressions, the brain (7 μm thick section) was covered with diluted (200×) antibodies targeting BLBP (Sigma Aldrich ABN14) and 4-HNE (Abcam, ab48506) for 16 hr in the cool and moist environment. The section was developed using fluorescent-tagged secondary antibodies Alexa Fluor^TM^ 405 (Thermo scientific, Waltham, MA, USA; A-31556) and Alexa Fluor^TM^ 594 (Invitrogen, Carlsbad, CA, USA, AB_2534073) against the primary antibodies of BLBP and 4-HNE, respectively. The IHC-stained brain section was visualized under fluorescent microscopy at the excitation and emission wavelength of 401/422 nm (for BLBP) and 590/618 nm (for 4-HNE).

### 4.11. Staining for Dihydroethidium (DHE), Acridine Orange (AO) and Cellular Senescence

In the brain DHE, and AO fluorescent staining were performed using the earlier-mentioned method [24]. Briefly, a tissue section (7 μm thick) was covered with 250 μL of DHE (30 μM) and AO (30 μg mL^−1^) solution followed by 5 min incubation, the section was rinsed with water, and visualized under the fluorescence microscope at the excitation and emission wavelength of 585/615 nm (for DHE) and 505/535 nm (for AO).

Brain cellular senesce was assessed by using senescence-associated-β-galactosidase (SA-β-gal) staining [24]. The brain section was covered with the 0.1% solution of 5-bromo-4-choloro-3-indolyl-β-D-galactopyranoside (X-gal) and incubated for 16 hr at RT. Following incubation, the stained section was examined under a microscope to identify senescent cells, which appeared blue.

### 4.12. Statistical Analysis

For the multiple comparisons, the statistical difference was evaluated by the One-way analysis of variance (ANOVA) following post-hoc analysis (Tukey’s), while for the pairwise comparisons, a two-tailed students *t*-test was utilized using the SPSS software (version 29.0; Chicago, IL, USA). A normality of distribution was performed on the data preceding the use of a parametric test.

## 5. Conclusions

Combined supplementation of banaba and policosanol exhibited markedly higher efficacy than their individual supplementation in mitigating HC+HG-induced dyslipidemia, oxidative stress, and restoration of plasma antioxidant parameters. Consistently, a significantly better effect was noticed on the tail fin regeneration, inhibition of hepatic inflammation, fatty liver changes, and protection of kidney and reproductive organs. In combination, banaba+policosanol (BNB+PCO) worked in a synergistic manner and ameliorated HC+HG-induced ROS production, apoptosis, and accumulation of BLBP, 4-HNE, and cellular senescence in the brain. The findings underscore the therapeutic potential of combined dietary intervention of banaba and policosanol in strengthening the antioxidant defenses, managing lipid profiles, and conferring the protection of organs from the external stress posed by HC+HG.

## Figures and Tables

**Table 1:** Comparative analysis of zebrafish survival and body weight (BW) changes under different 6-week dietary regimes.

	HC+HG	HC+HG +BNB	HC+HG +PCO	HC+HG +BNB+PCO
Survivability (%) at week 6	100	100	100	100
BW at week 0 (mg, *n* = 28)	299 ± 11	312 ± 10	323 ± 10	309 ± 10
BW at week 6 (mg, *n* = 28)	448 ± 16 ***	439 ± 17 ***	434 ± 12 ***	399 ± 13 ***^,†^
Net increase in BW (mg)	149 ± 16	127 ± 17	111 ± 12	90 ± 13 ^†^
Net increase in BW (%)	50	41	34	29 ^†^

HC, high cholesterol; HG, high galactose; BNB, banaba; PCO, policosanol; BW, body weight. Data were analyzed using a *t*-test between week 0 and week 6 BW in the column (*** *p* < 0.001). Data were analyzed by using one-way ANOVA followed by Tukey’s multiple comparisons with respect to the HC+HG group (in a row) (^†^ *p* < 0.05).

**Table 2:** Consumption of different formulated diets.

Dietary Components (mg)	HC+HG	HC+HG +BNB	HC+HG +PCO	HC+HG +BNB+PCO
Tetrabits (ND)	6.60	6.59	6.59	6.58
Cholesterol (final 4%, *w*/*w*)	0.40	0.40	0.40	0.40
Galactose (final 30%, *w*/*w*)	3.00	3.00	3.00	3.00
Banaba (final 0.1%, *w*/*w*)	0.00	0.01	0.01	0.01
Policosanol (final 0.1%, *w*/*w*)	0.00	0.00	0.01	0.01
Total mixture (mg)	10.00	10.00	10.00	10.00

HC, high cholesterol; HG, high galactose; ND, normal diet; BNB, banaba; PCO, policosanol.

## Data Availability

The original contributions presented in the study are included in the article and supplementary material, further inquiries can be directed to the corresponding author.

## Supplementary Materials

The following supporting information can be downloaded at: https://www.mdpi.com/article/10.3390/ph18060860/s1, Supplementary Table S1: A certificate of analysis of the used banaba extract. Supplementary Table S2: A certificate of analysis of the used policosanol. Supplementary Material Section S1: List of the used chemicals and reagents. Supplementary Method Section S2: Methodology for the quantification of plasma lipid profile and hepatic function biomarkers. Supplementary Method Section S3: Methodology for the quantification of plasma paraoxonase activity.
